# The Structural Lesions of the Skin, Their Pathology and Treatment

**Published:** 1869-10

**Authors:** 


The Structural Lesions of the Skin, their Pathology and Treatment. Illustrated. By HOWARD F. DAMON, A.M., M.D; Fellow Mass. Med. Soc.; Secretary Boston Obst. Soc.; Admitting Physician to the Boston City Hospital, and Physician to Department for Treatment of Skin Diseases among Out-Patients; Author of Photographs of Skin Diseases, The Neurosis of the Skin, etc., etc. Philadelphia: J. B. LIPPINCOTT & Co. 1869. Pp. 255; 8vo.

Almost every author upon skin diseases has seemed to find it necessary to invent a new system of classificatiom. Not to mention those of the two preceding generations, when the science had fairly to be founded, we have, at the present day, the widely different classification of Hebra, Devergie, Hardy, Cazenave, Wilson, and Buchanan; all eminent men, while others scarcely less eminent have introduced noted modifications into those systems which they have adopted as their basis.

Dr. Damon also claims "a new system of classification, based" as he says, "upon anatomical, physiological, and pathological data." "All skin diseases" he remarks, "may be separately included under one or more of the following causes:—secretion, nutrition, and structure,"—and thus he obtains the following classification:—

CLASS I.—Neurosis.
" II.—Functional Diseases of Cutaneous Glands.
" III.—Inflammation of the Skin.
" IV.—Structural Lesions of the Skin.

This awakens a shadowy recollection, as though familiar. We find Rayer's classification of 1835 to be—

CLASS I.—Inflammatory Affections.
" II.—Non-inflammatory Affections.
" III.—Diseases of the Secreting Functions.
" IV.—Neurosis.
" V.—Faulty Structure, or Unusual State of one or other of the Elementary Components of the Skin.

" VI.—Degenerations.

Now, if we omit the second of these as supernumerary, and condense the fifth and sixth into one, with a name which shall express their common character—say structural lesions—there results a classification, not merely similar, but identical with the one proposed by Dr. Damon. Although possessing hardly as much originality as the author claims, the classification is a good one, and a decided improvement on Rayer's.

Dr. Damon very naturally divides "structural lesions" into hypertrophies, atrophies, and pathological new formations (nutrition increased, diminished, or perverted). Comparing his with Hebra's similar divisions, we find that he has transferred to pathological new formations, lupus and elephantiasis arabum, the one from Hebra's "Chronic Exudata;" and the other from his hypertrophies. These changes seem very reasonable to us, in view of the recent investigations in the pathology of these diseases. On the other hand, callus, molluscum, and condyloma are transferred to hypertrophies, while the important subject of cicatrices is entirely omitted.

In his introduction, Dr. Damon has shown the scope of the work, and given brief notices of the more important lesions, with reasons for certain new views, so that a student will undertake the work understandingly.

The various hypertrophies are treated in a condensed way, most of them being passed by with few words.

The author recommends careful study of the position and form of callosities, as from them "it is quite possible, in many instances, to identify a person as belonging to a certain trade," and "it may be the subject of medico-legal evidence, in cases of doubtful identity."

Pigment excesses receive more attention than the other hypertrophies. From facts presented by other observers, Dr. Damon draws the conclusion, "that the deposition of pigment in the skin, under certain physiological and morbid conditions of the economy, depends upon the influence of the ganglionic nervous system." So far as presented, the data certainly seem to indicate this generalization. He does not bring this view forward as a new theory, but claims simply more or less to demonstrate what has hitherto been merely assertion. The article in this connection on Addison's disease, giving generalizations from 202 cases, is very interesting.

22 pages suffice for atrophies.

Pathological new formations derives its chief interest from the articles on elephantiasis arabum and elephantiasis grœcorum, which give in small compass the latest views upon these subjects. The descriptions of both diseases are lucid, and while showing resemblances, show, at the same time, obvious differences existing between them, both in appearance and in pathology. Dr. Damon regards "the origin and seat" of E. arabum to be "in the lymphatic vessels and glands of the affected extremity." The disturbance in these glands causes an interruption to the passage of lymph, and the subsequent phenomena." He quotes very concisely the opinions of Rasmussen and M. Mestre upon this disease, and agrees with the latter when he says: "two predispositions are necessary, in order that elephantiasis may become developed—one due to climateric action, and the other dependent on an idiosyncracy of the individuals."

The symptoms and appearance of E. grœcorum, Dr. Damon thinks, receive the most satisfactory explanation from the lesions found in the central nervous system, for the knowledge of which we are indebted to the investigations of Danielssen and Boeck. He regards it as hereditary, but not contagious, and thinks the light thrown on the therapeutics of diseases of the cerebro-spinal system, by the recent experiments of Brown-Séquard, will enable us to treat the disease understandingly, and with hope of benefit.

The work closes with a history of human horns, from A.D. 1590 to A.D. 1869, and a biography, which shows how extensive have been the researches of the author.

We commend the book to all interested in these subjects, as an honest exposition of the best views. The style is clear and beautifully concise. The publishers have done their part, also, to make the book a credit to any library. The beautiful paper, clear type, and wide margin give the eye satisfaction in its perusal.

We shall look with interest for the succeeding works.

